# Flow velocity quantification by exploiting the principles of the Doppler effect and magnetic particle imaging

**DOI:** 10.1038/s41598-021-83821-w

**Published:** 2021-02-25

**Authors:** Dennis Pantke, Florian Mueller, Sebastian Reinartz, Fabian Kiessling, Volkmar Schulz

**Affiliations:** 1 Department of Physics of Molecular Imaging, Institute for Experimental Molecular Imaging, RWTH Aachen University, Aachen, Germany; 2 Institute for Experimental Molecular Imaging, Medical Faculty, RWTH Aachen University, Aachen, Germany; 3 Department of Diagnostic and Interventional Radiology, Uniklinik RWTH Aachen, Aachen, Germany; 4 Fraunhofer Institute for Digital Medicine MEVIS, Bremen, Germany; 5 III. Physikalisches Institut B, RWTH Aachen University, Aachen, Germany

**Keywords:** Cardiology, Biomedical engineering, Techniques and instrumentation

## Abstract

Changes in blood flow velocity play a crucial role during pathogenesis and progression of cardiovascular diseases. Imaging techniques capable of assessing flow velocities are clinically applied but are often not accurate, quantitative, and reliable enough to assess fine changes indicating the early onset of diseases and their conversion into a symptomatic stage. Magnetic particle imaging (MPI) promises to overcome these limitations. Existing MPI-based techniques perform velocity estimation on the reconstructed images, which restricts the measurable velocity range. Therefore, we developed a novel velocity quantification method by adapting the Doppler principle to MPI. Our method exploits the velocity-dependent frequency shift caused by a tracer motion-induced modulation of the emitted signal. The fundamental theory of our method is deduced and validated by simulations and measurements of moving phantoms. Overall, our method enables robust velocity quantification within milliseconds, with high accuracy, no radiation risk, no depth-dependency, and extended range compared to existing MPI-based velocity quantification techniques, highlighting the potential of our method as future medical application.

## Introduction

Cardiovascular diseases (CVD) are the major cause of death worldwide^1^. Most types of CVD including ischemic stroke and myocardial infarction involve atherosclerosis. The gold standard to detect atherosclerosis is visualizing the atherosclerotic lesion using angiography, which is based on anatomic imaging modalities as X-ray, computed tomography (CT) or magnetic resonance imaging (MRI). Besides visualizing the luminal narrowing directly, the level of coronary plaque burden can also be estimated from measured blood flow velocities^2,3^. Other pathologies that can be assessed by blood flow information involve valvular defects or diastolic dysfunction^4,5^. Moreover, modeling pressure gradients and flow patterns is crucial for the design and planning of bypass and graft surgeries^6,7^.

Techniques to measure blood flow velocities that are clinically available include phase-contrast MRI, X-ray digital subtraction angiography (DSA) and Doppler-ultrasound. However, each approach has its shortcomings: X-ray DSA comes with high temporal and spatial resolution, but uses ionizing radiation^8,9^. Phase-contrast MRI is free of ionizing radiation, however, three-dimensional (3D) velocity imaging is time-consuming and suffers from respiratory motion artifacts^10–12^. Doppler-ultrasound is able to perform real-time measurements, but suffers from user dependence, angle sensitivity and the reliability decreases with the depth from the patient’s surface^8,13^. A highly accurate, non-invasive, radiation-free, depth-independent and fast modality is still of great interest.

Magnetic particle imaging (MPI) is a novel tracer-based imaging modality that promises to overcome these limitations. MPI determines the spatial distribution of super-paramagnetic iron oxide-based nanoparticles (SPIONs) by exploiting their non-linear magnetization function^14–16^. Signal generation is performed by applying dynamic magnetic (drive) fields and measuring the particles’ magnetization response. Spatial encoding is enabled by applying a constant magnetic field gradient (selection field), characterized by a field free point (FFP), which defines the origin of signal generation. In addition to the mentioned properties, MPI features sub-millimeter resolution^17,18^ and a high sensitivity shown by a reported detection limit of 5–20 ng(Fe)^19–21^.

Due to its high time resolution with a repetition time $T_{\mathrm{R}}$ of 21.5 ms resulting in 46 volumes per second^15^ (which can be even higher dependent on the excitation field frequency), magnetic particle imaging is able to quantify blood flow velocities by tracking the tracer bolus in the image space^22–27^. Therefore, a series of images has to be acquired with high temporal resolution. From the time interval between the consecutive images and the time- and position-dependent signal intensities in the images, the bolus velocity can be estimated. Flow velocity quantification from image space is a robust approach to track the velocity of SPION boluses, however, the maximum velocity that can be quantified is restricted by the field of view (FOV) in direction of flow $x_\text{FOV}$ and the time resolution of the employed scanner. The upper velocity detection limit is reached, if the bolus is too fast to be imaged in two consecutive images. This is the case, if the distance that the bolus travels during one imaging repetition exceeds the field of view. Thus, the maximum velocity $v_\text{x}^\text{max}$ that can be detected by image-based velocity quantification is:

1 $$\begin{aligned} v_\text{x}^\text{max} = \dfrac{x_\text{FOV}}{T_\text{R} } . \end{aligned}$$

The estimated velocities by existing MPI-based velocity quantification techniques do not exceed 0.4 m/s^27^ and 1.0 m/s^26^ while the repetition times $T_\text{R}$ of the image acquisitions were 20 ms and 21.54 ms, respectively. Moreover, the estimated velocities by image-based velocity quantification depend on which image reconstruction technique and parameters as well as velocity estimation technique is used.

In this work, we propose a novel method that uses the principle of magnetic particle imaging and spectroscopy (MPS; no spatial encoding) to quantify flow velocities based on the raw frequency spectrum. The method makes use of the well known Doppler effect^28^, which was adapted to MPI: due to the nanoparticles’ motion in the MPI’s laboratory reference system, the FFP trajectory in the SPIONs’ reference frame is modulated. Consequently, the magnetization response of the nanoparticles is modulated as well, which results in a frequency shift of the particles’ signal manifesting in a split-up of the higher harmonics in frequency space. The frequency shift is a function of the tracer’s velocity and can therefore be used for velocity quantification.

In this manuscript, the basic theory of Doppler-MPS is presented, followed by simulations illustrating the frequency shift caused by the MPI signal modulation. Finally, phantom measurements that mimic uniform linear flow and flow velocity reconstructions using the Doppler-MPS technique are demonstrated.

**Figure 1:**
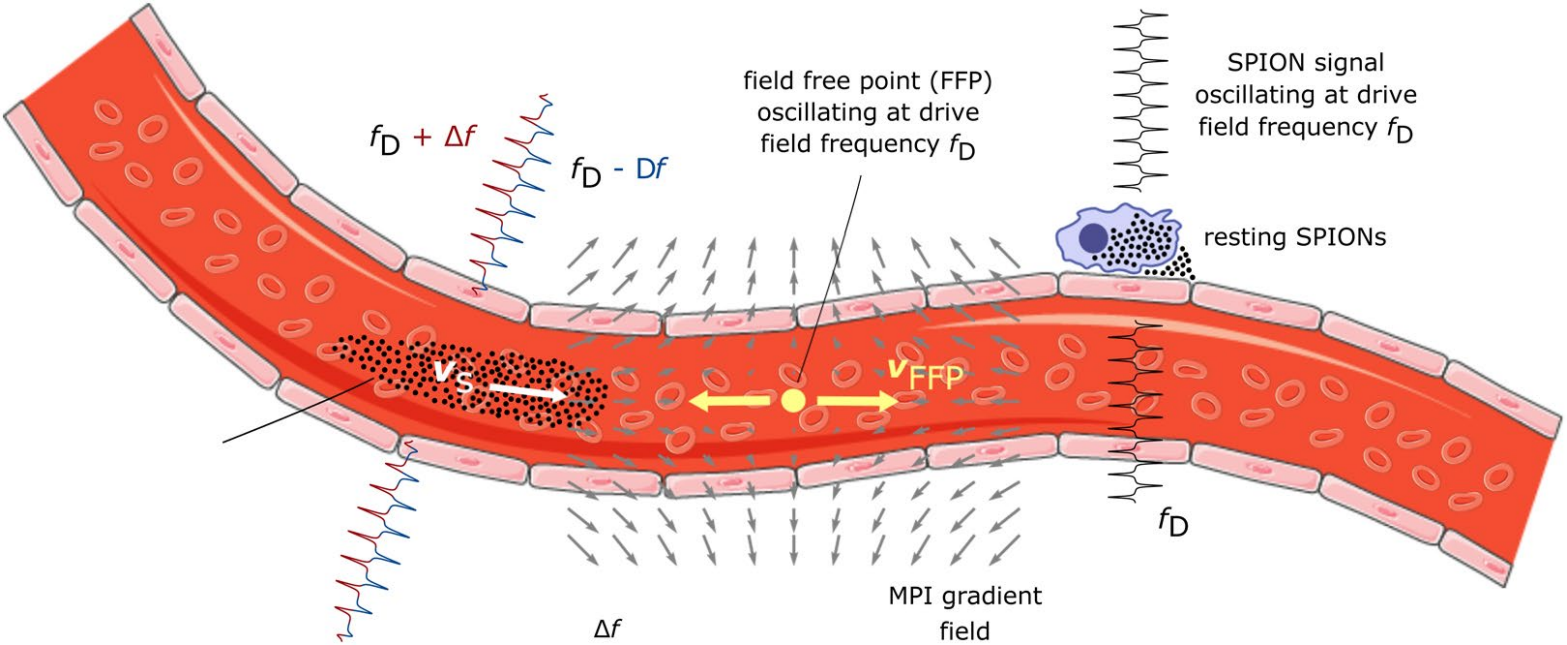
Illustration of the Doppler-MPS working principle. The moving SPIONs are exposed to the oscillating homogeneous drive field, superimposed by the selection field, which features a field free point (FFP) in the center. While the FFP is oscillating at the drive field frequency $f_\text{D}$ in one dimension, the signal emitted by the moving SPIONs exhibits a frequency shift $f_\text{D} \pm \Delta f$, which is a function of the SPION velocity $v_{\mathrm{s}}$ and can therefore be exploited for velocity quantification. The signal emitted by resting particles is oscillating at the drive field frequency $f_\text{D}$ and thus can be distinguished from the signal emitted by moving particles. (The image was built upon material from Servier Medical Art (http://smart.servier.com), which is licensed under Creative Common Attribution 3.0 Unported License (CC BY 3.0), https://creativecommons.org/licenses/by/3.0/).

**Figure 2:**
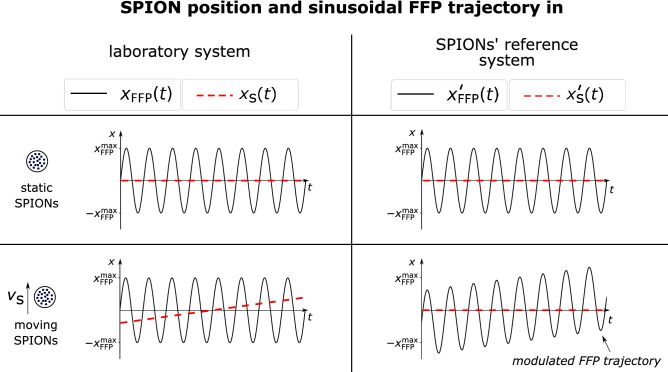
Field free point (FFP) trajectory modulation. Temporal progress of FFP and SPION position in laboratory system ($x_{\mathrm{FFP}}(t), x_{\mathrm{s}}(t)$) and reference frame of the nanoparticles ($x_{\mathrm{FFP}}^{\prime }(t), x_{\mathrm{s}}^{\prime }(t)$) for static and moving SPIONs. The modulation of the FFP trajectory in the SPIONs’ reference system $x_{\mathrm{FFP}}^{\prime }(t) = x_{\mathrm{FFP}}(t) + v_{\mathrm{s}} t$ is caused by the particles’ motion in the laboratory system.

**Figure 3:**
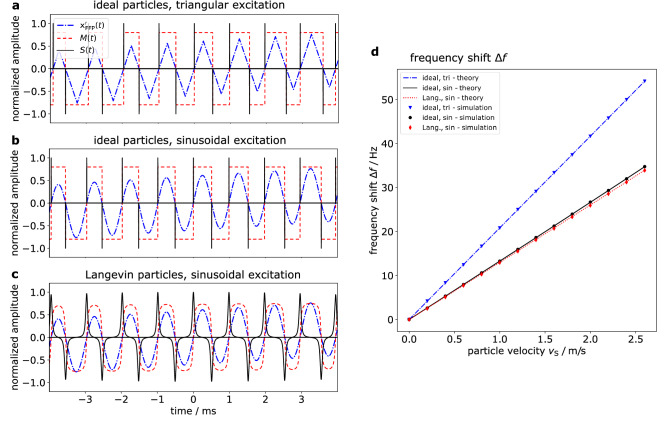
Signal trajectories for moving particles and frequency shift against particle velocity for different particle types and excitation waveforms. Simulations of the FFP trajectory $x_{\mathrm{FFP}}^{\prime }(t)$, the particles’ magnetization *M*(*t*) and the signal *S*(*t*) are shown for three different cases: (**a**) Ideal particles and triangular excitation, (**b**) ideal particles and sinusoidal excitation, (**c**) Langevin particles ($m_0 = 3.35 \cdot 10^{-18}$ Am$^2$, *T* = 293 K) and sinusoidal excitation. The trajectories are shown for moving particles at 1.5 m/s. The signal, magnetization and FFP trajectory were normalized to 1, 0.8 and 0.5, respectively, with the purpose to better distinguish between the curves. The drive field frequency was exemplarily set to 1 kHz. In case of ideal particles, the delta peaks in the signal appear during zero crossings of the FFP trajectory. (**d**) Shift of the signal’s fundamental frequency $\Delta f$ over the particles’ velocity $v_{\mathrm{s}}$ for three different cases derived theoretically and based on simulation: Ideal particles and triangular excitation (blue, dashdotted line; triangles), ideal particles and sinusoidal excitation (black, solid line; dots), Langevin particles ($m_0 = 3.35 \cdot 10^{-18}$ Am$^2$, *T* = 293 K) and sinusoidal excitation (red, dotted line; diamonds). The amplitude of the FFP trajectory is $x_{\mathrm{FFP}}^\text{max} = 12$ mm.

## Results

### Basic principle of Doppler-MPS

The working principle of Doppler-MPS is illustrated in Fig. 1. The magnetization response of resting SPIONs, which are periodically driven into saturation by the superposition of the drive field—oscillating one-dimensionally at the frequency $f_\text{D}$—and the selection field, features a fundamental harmonic at $f_\text{D}$ and its higher harmonics in frequency space. In contrast, if the particles move in the laboratory system while being exposed to the time-varying magnetic field, their magnetization response will exhibit a frequency shift $f_\text{D} \pm \Delta f$, which depends on the particles’ velocity and can therefore be used for velocity quantification.

The frequency shift of the fundamental harmonic $\Delta f$ as a function of the particles’ velocity $v_{\mathrm{s}}$ in the laboratory system were derived mathematically. A detailed derivation of the frequency shifts as a function of the particle velocity is provided in the Methods section. The derivation is based on the principle that SPION motion in the laboratory system causes a modulation of the FFP trajectory in the SPIONs’ reference system $x_{\mathrm{FFP}}^{\prime }(t)$. This is illustrated in Fig. 2. The modulated FFP trajectory in the particles’ reference system can be written as

2 $$\begin{aligned} x_{\mathrm{FFP}}^{\prime }(t) = x_{\mathrm{FFP}}(t) + v_{\mathrm{s}} t, \end{aligned}$$

where $x_{\mathrm{FFP}}(t)$ is the FFP trajectory in the laboratory system. The frequency shifts were derived for three different cases varying in particle type and excitation waveform: (1) ideal particles, triangular excitation (2) ideal particles, sinusoidal excitation (3) Langevin particles, sinusoidal excitation. In section Methods: Derivation of frequency shift, it is shown that for case (1) the frequency shift $\Delta \omega = 2 \pi \Delta f$ of the fundamental harmonic is independent of the excitation frequency $f _\text{D}$:

3 $$\begin{aligned} \Delta f = \frac{v_{\mathrm{s}}}{4 \, x_{\mathrm{FFP}}^\text{max}} . \end{aligned}$$

For the total shifted fundamental frequency $f_{\mathrm{s}}$, one gets two solutions according to increasing, respectively, decreasing FFP trajectory. As a consequence, the harmonics in frequency space split up into two peaks. The shifted frequency can be calculated according to $f_{\mathrm{s}} = f_\text{D} \pm \Delta f$. The respective higher harmonics of the signal are at $f_{\mathrm{s} \, \mathrm{n}}=n \cdot f_{\mathrm{s}}$ and the frequency shift of a specific harmonic is $\Delta f_\text{n} = n \cdot \Delta f$ with $n \in \mathbb {N}$.

For each of the abovementioned cases, the modulated FFP trajectory in the particles’ reference system $x_{\mathrm{FFP}}^{\prime }(t)$, the particles’ magnetization response *M*(*t*) and the MPI signal *S*(*t*) (which is the magnetization response induced into receive coils) are depicted in Fig. 3a–c. The drive field is exemplarily set to 1 kHz and the signals are normalized to 0.5 for $x_{\mathrm{FFP}}^{\prime }(t)$, to 0.8 for *M*(*t*) and to 1 for *S*(*t*), to better distinguish between the curves. For case (3), Langevin particles at room temperature (*T* = 293 K) with a single magnetic moment $m_0 = 3.35 \cdot 10^{-18}$ Am$^2$ are assumed. The derived frequency shifts are plotted in Fig. 3d. For the cases of sinusoidal excitation, the period time of the particle signal $T_{\mathrm{s}}$ is slightly changing with the number of FFP oscillation periods *n*. Therefore, the frequency shifts were calculated based on the first period of the particle signal. For the calculation of the frequency shifts, an exemplary FFP amplitude of $x_{\mathrm{FFP}}^\text{max} = 12$ mm was assumed.

For all three cases, the frequency shift increases linearly with the particles’ velocity in the observed velocity range from 0 m/s to 2.6 m/s, while triangular excitation and ideal particles leads to the highest frequency shifts followed by sinusoidal excitation and ideal particles. The frequency shifts resulting from sinusoidal excitation and Langevin particles are only slightly lower than for the case of sinusoidal excitation and ideal particles. The theoretical observations indicate that the proposed approach is a quantitative tool to determine flow velocities.

**Table 1:** Validation of theoretically derived frequency shifts by simulation. Mean and standard deviation of the absolute differences $\mid \overline{\Delta f_\text{theo}(v_{\mathrm{s}})} - \overline{\Delta f_\text{sim}(v_{\mathrm{s}}})\mid$ of derived frequency shifts by theory and simulation.

Particle type/excitation	Mean absolute difference (Hz)	Standard deviation (Hz)
Ideal/triangular	0.0029	0.0315
Ideal/sinusoidal	0.0005	0.0272
Langevin/sinusoidal	0.0519	0.0267

### Simulations

**Figure 4:**
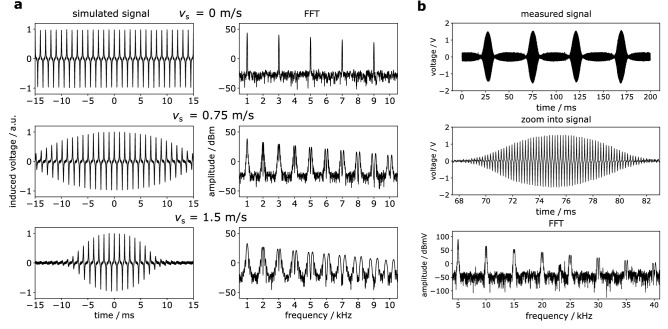
Simulated and measured data in time and frequency domain. (**a**) Simulated signal of a delta sample performing uniform linear motion in time and frequency domain for different sample velocities: $\lbrace 0, 0.75, 1.5\rbrace$ m/s. The simulation parameters were set to: $f_\text{D} =$ 1 kHz, $x_{\mathrm{FFP}}^\text{max} = 12$ mm, $G = 1$ T/m, $m_0 = 3.35 \cdot 10^{-18}$ Am$^2$. The higher harmonics in frequency space split up into two peaks for moving particles. (**b**) Measured signal of the oscillating Perimag sample at the analog-to-digital converter (ADC) in time (top, center) and frequency space (bottom). The signal is shown exemplarily for an excitation frequency of 5 kHz and a quantified sample velocity of 2.07 m/s. In time domain, four field of view (FOV) crossings are shown (top) and a zoom into one FOV crossing is provided (middle). To obtain the signal in frequency space, the Fourier transform of the signal caused by only one FOV crossing is performed. The splitting of the higher harmonics due to the sample motion can be seen in the frequency spectrum, particularly at high harmonic numbers.

**Figure 5:**
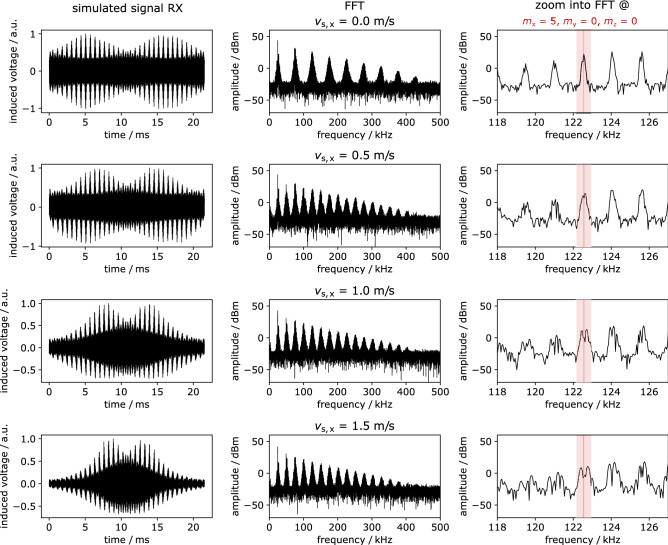
Simulated signal of moving tracer scanned by 3D-Lissajous FFP trajectory. Signal induced into the Rx-channel (receive coil is sensitive to magnetization changes in *x*-direction) of uniformly moving sample in *x*-direction in time (left) and frequency domain (center, right) for different sample velocities: $\lbrace$0, 0.5, 1.0, 1.5$\rbrace$ m/s. The drive field frequencies are $f_\text{x}$ = 24.5098 kHz, $f_\text{z}$ = 26.04167 kHz and $f_\text{z}$ = 25.2525 kHz, resulting in a 3D Lissajous trajectory with a repetition time of 21.55 ms as implemented in the preclinical MPI Bruker 25/50 (Bruker BioSpin GmbH, Ettlingen, Germany). The other relevant simulation parameters were as well aligned to the Bruker device: $H_{\mathrm{D} \, \mathrm{x,y,z}} = \mathrm {14 \, \, mT/\mu _0}$, $G_\text{x}$ = 2.5 T/m, $G_\text{y,z} = \frac{1}{2}G_\text{x}$, $x_{\mathrm{FFP}}^\text{max}$ = 5.6 mm, $y_{\mathrm{FFP}}^\text{max}$ = $z_{\mathrm{FFP}}^\text{max}$ = 11.2 mm, $m_0 = 3.35 \cdot 10^{-18}$ Am$^2$, *T* = 293 K. The images on the right show that the frequency splitting is visible for sample velocities unequal to zero at the frequency component resulting from the mixing factors $m_\text{x}$ = 5, $m_\text{y}$ = 0 and $m_\text{z}$ = 0.

**Figure 6:**
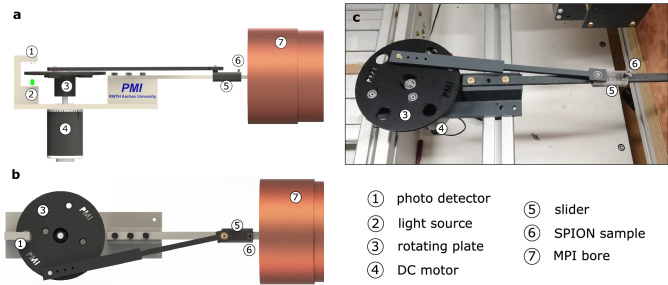
Custom designed mechanical assembly enabling the oscillation of a SPION sample inside the MPI bore. (**a**) CAD design, side view. (**b**) CAD design, top view. (**c**) Photography of manufactured assembly. The sample (6) is fixed on a slider (5) that is connected to a rotating plate (3) via a stiff rod. The rotating plate is driven by a DC motor (4), which is connected to a pulse-width modulation rotational speed controller. Calibration of “ground truth” sample velocity is performed by measuring the rotation frequency of the rotating plate. The rotation period is determined from the time difference between two consecutive light pulses (2) that are sensed by a photo detector (1).

Qualitative simulations of the particle signal that would be seen by an MPI receive coil were performed to validate the theoretically derived frequency shifts $\Delta f$ as a function of the particles’ velocity $v_{\mathrm{s}}$. Again, the three aforementioned cases were considered. In case of Langevin particles, the magnetization *M*(*t*) is calculated according to

4 $$\begin{aligned} M(t) = c \, m_0 \, \mathcal {L}\left( \frac{\mu _0 m_0}{k_\text{B} T} \, H^{\prime }(t)\right) . \end{aligned}$$

While *c* is the particle concentration, $m_0$ denotes the magnitude of a single particle’s magnetic moment, *T* the temperature, $\mu _0$ the vacuum permeability and $k_\text{B}$ is Boltzman’s constant^16,29^. The Langevin function is defined as

5 $$\begin{aligned} \mathcal {L}(\xi ) := {\left\{ \begin{array}{ll} \mathrm{coth}(\xi ) - 1 / \xi &{}\mathrm{for} \ \xi \ne 0 \\ 0&{}\mathrm{for} \ \xi = 0 \end{array}\right. } \, . \end{aligned}$$

The magnetization of ideal particles is calculated based on

6 $$\begin{aligned} M(t) = c \, m_0 \, \mathrm{sgn}\left( \frac{\mu _0 m_0}{k_\text{B} T}\, H^{\prime }(t)\right) , \end{aligned}$$

where $\mathrm{sgn}(H)$ is the sign-function, which is defined as

7 $$\begin{aligned} \mathrm{sgn}(H)= {\left\{ \begin{array}{ll} 1&{}\mathrm{for} \,H > 0\\ 0&{}\mathrm{for}\, H = 0\\ -1&{}\mathrm{for} \,H < 0\\ \end{array}\right. } \end{aligned}.$$

The MPI signal *S*(*t*) was determined by numerically calculating the derivative of the magnetization and adding gaussian noise. Since for the purpose of determining the frequency shifts, a quantitative simulation of the induced voltages is not required, other components of the receive chain as low-noise amplifier (LNA), filter or coil sensitivity are not taken into account. Relaxation effects are not considered. To ensure comparability with the mathematical derivation, the frequency shifts are determined according to first period of the simulated time domain signal with following simulation parameters: $x_{\mathrm{FFP}}^\text{max} = 12$ mm, $G = 1$ T/m, $m_0 = 3.35 \cdot 10^{-18}$ Am$^2$, *T* = 293 K.

The frequency shifts based on the simulations are plotted together with the theoretically derived frequency shifts in Fig. 3d. As already stated in the previous section, the frequency shift increases linearly with the particles’ velocity in the observed velocity range from 0 m/s to 2.6 m/s, while triangular excitation and ideal particles leads to the highest frequency shifts followed by sinusoidal excitation and ideal particles. This is validated by the simulated data. The relative difference of the simulated frequency shifts at 1 m/s between cases (1) and (3) is 60.6 % but only 2.4 % between cases (2) and (3).

The results determined from the simulated data and the theoretically derived results match very well for all observed cases. The mean absolute difference $\mid \overline{\Delta f_\text{theo}(v_{\mathrm{s}})} - \overline{\Delta f_\text{sim}(v_{\mathrm{s}}})\mid$ is always $<0.1$ Hz. The mean differences and standard deviations for the three respective cases can be taken from Table 1.

The simulated signal is shown for the sample velocities of 0 m/s, 0.75 m/s and 1.5 m/s in Fig. 4a. To make the frequency shifts visible in frequency space, fast Fourier transforms (FFT) were performed on the simulated time domain data. In case of the non-moving sample, the characteristic MPI signal can be observed, which shows peaks at odd harmonics of the fundamental frequency, since the particles are positioned at the center of the selection field. For the case of moving particles, the signal is generated when the sample passes the FOV, which is the region of particle excitation. Here, the splitting of the higher harmonics can be observed. In the frequency spectrum, the higher harmonics of the samples moving with 0.75 m/s and 1.5 m/s can be separated into two peaks.

To prove the feasibility of Doppler-MPS for the case that a 3D drive field is applied, simulations of a moving delta sample were performed, in which the FFP is driven on a 3D-Lissajous trajectory by the drive field frequencies $f_\text{x}$ = 24.5098 kHz, $f_\text{y}$ = 26.04167 kHz and $f_\text{z}$ = 25.2525 kHz. The resulting repetition time is $T_\text{R}$ = 21.5 ms. Other relevant simulation parameters are: $H_{\mathrm{D} \, \mathrm{x,y,z}} = {14 \, \, \mathrm{mT}/\mu _0}$, $G_\text{x}$ = 2.5 T/m, $x_{\mathrm{FFP}}^\text{max}$ = 5.6 mm, $m_0 = 3.35 \cdot 10^{-18}$ Am$^2$, *T* = 293 K. To visualize the peak splitting in frequency space, the signal of a delta sample moving with $v_{\mathrm{s} \, \mathrm{x}} = \lbrace 0, 0.5, 1, 1.5\rbrace$ m/s was simulated. To determine the velocity-dependent frequency shifts, the velocity $v_{\mathrm{s} \, \mathrm{x}}$ was varied between 0 m/s and 2.6 m/s in 0.2 m/s steps. The impact of shifting the sample motion trajectory in parallel to the central axis of the FOV is investigated.

Figure 5 shows the signal that is induced into the receive coil that is most sensitive to magnetization changes in *x*-direction (Rx-channel) in time (left) and frequency space (center, right). On the right, a zoom into the frequency spectrum is shown to visualize the splitting of the frequency component resulting from the mixing factors $m_\text{x}$ = 5, $m_\text{y}$ = 0 and $m_\text{z}$ = 0: $f_\text{mix} = m_\text{x} f_\text{x} + m_\text{y} f_\text{y} + m_\text{z} f_\text{z} = 122.6$ kHz. The splitting of this specific peak is visible for all sample velocities unequal to zero. Supplementary Fig. S1a shows the frequency shift against the particle velocity for different distances $y_\text{sample}$ between the parallel trajectories of the particle sample and the FFP for 1D excitation. The frequency shift decreases for an increasing distance $y_\text{sample}$. In supplementary Fig. S1b, the slope of the frequency shifts’ linear regression is drawn against the distance $y_\text{sample}$. In case of 1D sampling, the frequency shift decreases with increasing distance $y_\text{sample}$, while for 3D sampling the frequency shift is almost constant. The frequency shift against the sample velocity $v_{\mathrm{s} \, \mathrm{x}}$ determined from the 3D simulation compared to the 1D simulation is plotted in supplementary Fig. S1c. The frequency shift for a 3D-Lissajous FFP trajectory is decreased compared to 1D excitation. The respective linear regressions of the frequency shift as a function of the velocity are $\Delta f = 31.01 v_{\mathrm{s} \, \mathrm{x}} - 0.05$ (1D FFP trajectory) and $\Delta f = 22.63 v_{\mathrm{s} \, \mathrm{x}} + 0.34$ (3D-Lissajous FFP trajectory). The frequency shift was determined by analyzing the frequency components resulting from the mixing factors $m_x = \lbrace 4,5,6\rbrace , m_y = 0, m_z = 0$ and averaging the determined frequency shift.

### Measurement setup

To validate the simulation results and present first velocity quantifications, measurements of a moving SPION sample were performed at the in-house developed multi-frequency scanner (mf-MPI)^30^. The one-dimensional (1D) mf-MPI offers an adjustable excitation frequency in the range of 0.5 to 20 kHz, drive field amplitude up to 10 mT and the detection of the fundamental harmonic.

Uniform, linear motion is mimicked by an oscillating particle sample in parallel to the FFP trajectory, but with greater amplitude. Thus, the measurement setup parameters are highly controllable and measurements are easily repeatable. The sample oscillation is enabled by a custom designed, MPI-compatible mechanical assembly depicted in Fig. 6. All components inside the MPI bore are non-magnetic and non-conductive. The sample is fixed on a slider that is connected to a rotating disc via a stiff rod. The connections disc-rod and rod-slider are realized via non-magnetic ball bearings. The whole mechanical assembly is centered in front of the MPI bore by an assembly of aluminum profiles.

The rotating plate is driven by a DC motor, connected to a pulse-width modulation rotational speed controller. Thus, the input voltage to the DC motor is controllable, however, due to friction in the mechanical assembly, the rotational speed can only be roughly estimated. Since the rotational speed is needed to calculate the “ground truth” sample velocity, the rotation frequency of the disc was determined by a setup that is composed of a light source and a photo detector. The period time is determined from the time difference between two consecutive light pulses that pass a hole in the disc and are sensed by the photo detector.

### Measurement parameters

The smallest flow velocity that can be quantified by Doppler-MPS depends on the spectral resolution $\Delta f_\text{sp}$, since the higher harmonics need to be separable to determine the frequency shift. The spectral resolution can be calculated according to

8 $$\begin{aligned} \Delta f_\text{sp} = \frac{s}{n_{\mathrm{s}}} = \frac{1}{t_\text{meas}}, \end{aligned}$$

where *s* is the sampling rate, $n_{\mathrm{s}}$ is the number of sample points and $t_\text{meas}$ the effective measurement time. To show the effect of spectral resolution, the experiments were conducted with two different sets of paramters:

1. $f_\text{D} = 2$ kHz, $t_\text{meas}$ = 250 ms, *s* = 0.5 MHz, $n_{\mathrm{s}}$ = 125000, $\Delta f_\text{sp}$ = 4 Hz.
2. $f_\text{D} = 5$ kHz, $t_\text{meas}$ = 200 ms, *s* = 1 MHz, $n_{\mathrm{s}}$ = 200000, $\Delta f_\text{sp}$ = 5 Hz.

The drive field amplitude is set to 7.5 mT in both sets. Since the gradient field is 0.7 T/m in *x*-direction, the resulting length of the FOV (distance that is passed by the FFP) is 21.4 mm. The peak-to-peak distance of the sample oscillation was 60 mm to ensure a nearly uniform motion in the boundaries of the FOV.

As there is no rotational speed control of the DC motor in the experimental setup, the DC input was varied to achieve different sample velocities. During the experiments with 5 kHz excitation, the DC input of the motor was varied from 1.56 V to 3.72 V (determined “ground truth” velocity: 0.80 m/s to 2.06 m/s) in 0.12 V steps. In case of the measurement with 2 kHz excitation, the voltage input was varied from 0.96 V (0.27 m/s) to 1.92 V (1.04 m/s), since the quantification of lower flow velocities should be shown. For each input voltage, 5 measurements were performed for averaging.

**Figure 7:**
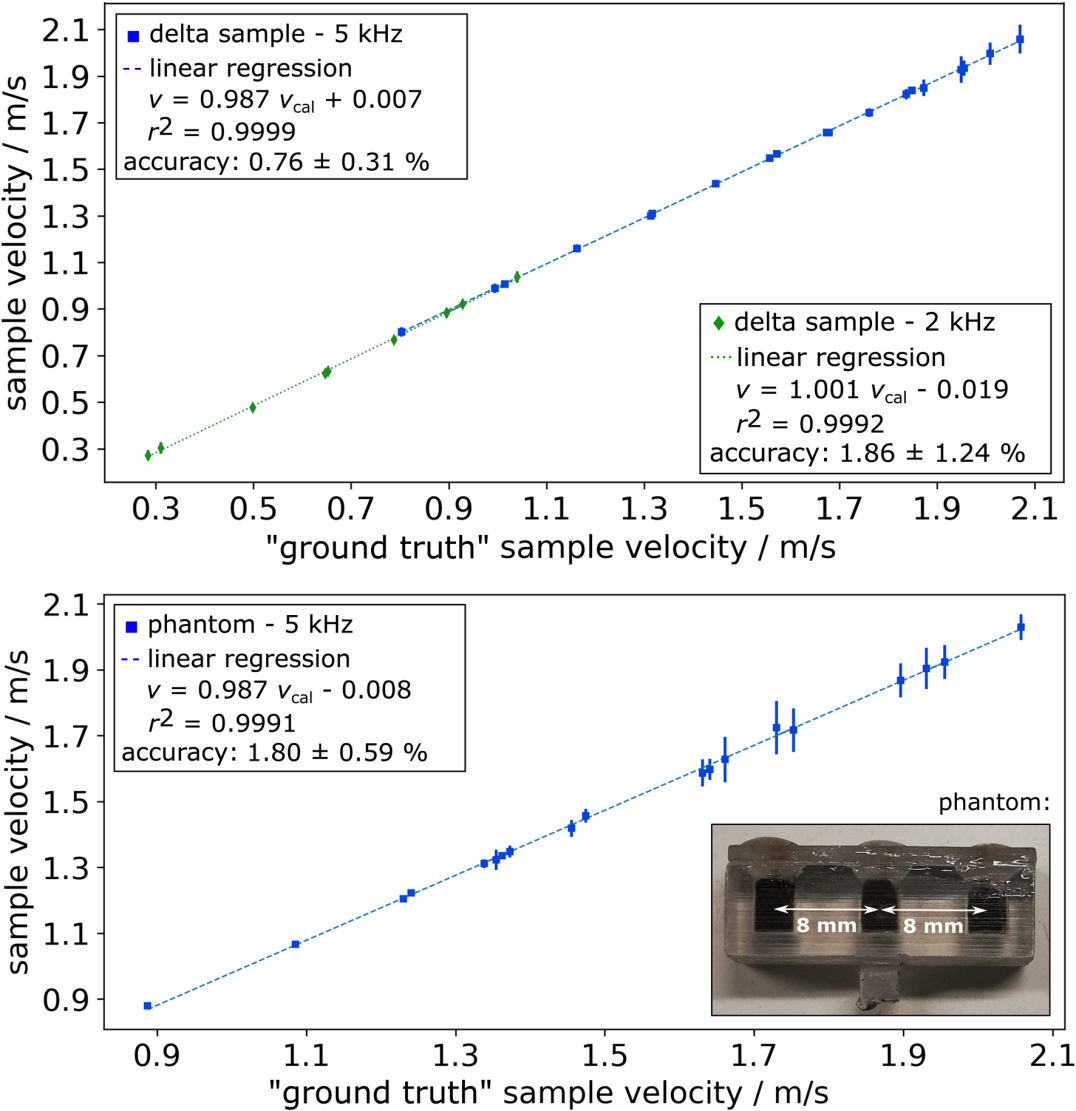
Flow velocity quantification. Quantified velocities plotted against the “ground truth” velocities of the oscillating Perimag sample at 2 kHz and 5 kHz excitation (top) and velocity quantifications of the oscillating 3-sample phantom at 5 kHz excitation (bottom). The linear equation and correlation coefficient $r^2$ of linear regression as well as the mean accuracy of each measurement series can be taken from the boxes at the corners of the figures. The phantom comprising three Perimag samples is shown bottom right. The error bars indicate the standard deviation of the quantified sample velocities. The velocity quantification for a specific input voltage of the DC motor was repeated five times in sequence and averaged.

### Delta sample measurement

The sample vessel on the slider was filled with 50 $\upmu$l of Perimag (micromod Partikeltechnologie GmbH, Rostock, Germany), which are clustered core particles with a hydrodynamic diameter of 130 nm showing good signal performance in MPI similar to Resovist^31^, which is clinically approved for liver diagnostic. The iron concentration was 8.5 mg/ml.

The signal of the oscillating sample in time and frequency domain is exemplarily shown for a sample velocity of 2.07 m/s at 5 kHz excitation in Fig. 4b, top. A signal is always generated when the sample crosses the field of view. The Fourier transform is performed on the signal resulting from only one FOV crossing by using a window function (Fig. 4b, center). As already seen in the simulated spectra, the higher harmonics split up in case of moving SPIONs (Fig. 4b, bottom).

The results of the flow velocity quantifications at 2 kHz and 5 kHz, respectively, can be seen in Fig. 7, top. The quantified velocity is the average velocity of the sample inside the FOV, which is almost constant, since the sample motion is almost uniform. The quantified sample velocities are plotted against the “ground truth” sample velocities (determined from rotation frequency). The mean accuracy was calculated and a linear regression was applied to assess the quality of the quantification. The accuracy is defined as the ratio of the absolute difference between the quantified sample velocity $v_{\mathrm{s}}^\text{q}(V_\text{in})$ and the “ground truth” sample velocity $v_{\mathrm{s}}^\text{gt}(V_\text{in})$ to the “ground truth” velocity at a specific input voltage of the DC motor $V_\text{in}$:

9 $$\begin{aligned} \mathrm{accuracy} := \frac{\mid v_{\mathrm{s}}^\text{gt}\left( V_\text{in}\right) -v_{\mathrm{s}}^\text{q}\left( V_\text{in}\right) \mid }{v_{\mathrm{s}}^\text{gt}\left( V_\text{in}\right) } . \end{aligned}$$

For both sets of parameters, there is a strong linear correlation between the quantified and “ground truth” sample velocities. In case of 5 kHz excitation, the linear equation is $v = 0.987 v_{\mathrm{cal}} + 0.007$, the correlation coefficient $r^2$ is 0.9999 and the mean accuracy is 0.76 ± 0.31 $\%$. The range of quantified sample velocities is between 0.80 m/s and 2.07 m/s. With 2 kHz excitation, the linear equation is $v = 1.008 v_\text{cal} - 0.019$, $r^2$ is 0.9992 and the achieved mean accuracy is 1.86 ± 1.24 $\%$. Flow velocities between 0.28 m/s and 1.04 m/s were measured.

### 3-sample phantom measurement

To show the ability of Doppler-MPS to quantify velocities of other particle distributions than delta samples, a phantom vessel was designed and 3D-printed by stereolithography (Fig. 7, bottom right). The prepared phantom is comprised of three cubic samples that contain 60 $\upmu$l Perimag with an iron concentration of 8.5 mg/ml. The distance between the samples is 8 mm (center-to-center) in *x*-direction and the dimensions of the samples are $\lbrace \Delta x,\Delta y,\Delta z\rbrace$ = $\lbrace$3,4,5$\rbrace$ mm. The voltage input of the DC motor was varied between 1.56 V (0.89 m/s) and 3.72 V (2.06 m/s) in 0.12 V steps. The drive field frequency was set to 5 kHz, the sampling rate was 1 MHz and the number of sample points were 200000.

Supplementary Fig. S2 shows the measured raw signal of the 3-sample phantom in time and frequency domain. Figure 7, bottom, shows the results of the velocity quantification. A photo of the phantom is shown bottom right. The range of quantified sample velocities here is between 0.89 m/s and 2.06 m/s. The quantified and “ground truth” sample velocities again show a strong linear correlation. The linear equation is $v = 0.987 v_\text{cal} - 0.008$, $r^2$ is 0.9991 and a mean accuracy of 1.80 ± 0.59 $\%$ was reached. Flow velocities between 0.88 m/s and 2.03 m/s were quantified.

## Discussion

In this manuscript, a novel approach to quantify flow velocities with magnetic particle imaging by analyzing the frequency shift caused by the motion of SPIONs has been presented. We called the method Doppler-MPS due to the overlap of the underlying principle with the Doppler effect.

The basic principle of Doppler-MPS was described and the SPION signal’s frequency shift depending on the particle velocity was derived mathematically. Qualitative simulations of the signal emitted by a uniformly moving delta sample as a response to one-dimensional sinusoidal excitation have been performed. The simulation could validate the theoretically derived frequency shifts as a function of the sample velocity for three different cases: (1) ideal particles, triangular excitation (2) ideal particles, sinusoidal excitation (3) Langevin particles, sinusoidal excitation (cf. Fig. 3d, Table 1). In all cases, the frequency shift seems to increase linearly with the particle velocity. However, this only holds true for case (1) as also can be taken from Eq. (19). For cases (2) and (3), the frequency shift increases almost linearly in the observed range of velocities 0 m/s to 2.6 m/s. For even higher velocities, there will be a non-linear increase, since the period time of the particle signal is proportional to the intercept of the (non-linear) sinusoidal FFP trajectory and the particles’ trajectory.

The particle properties as well as the drive field waveform affect the frequency shift and need to be considered when performing a calibration (based on the theoretical model, simulation or measurements) of the frequency shifts depending on the sample velocities for later flow velocity quantification. The slope of the Langevin function’s dynamic region increases with core size until the sign-function represents the ideal case. However, since the difference of the frequency shift between ideal and Langevin particles is rather small (Fig. 3d), the method is quite robust against improper knowledge of the tracer core size. The relaxation dynamics of the particles, which was neglected during the simulations, is affected by particle properties as anisotropy, hydrodynamic volume and core size. Relaxation can be modeled as convolution with a low-pass relaxation kernel, which translates in an attenuation of higher harmonics in frequency domain, thus, a major affect of the relaxation on the frequency shift is not expected. Potentially, the lower detection limit of a shifted harmonic can be affected by the mentioned parameters, since the SNR of higher harmonics is reduced for increased relaxation time.

Figure 4a shows the split-up of higher harmonics in the frequency spectrum based on the simulations. Finally, the principle of Doppler-MPS, i.e. the frequency shift caused by the modulation of the FFP trajectory in the reference frame of the particles, was derived theoretically and validated in simulation. The related effect of harmonic dispersion as a consequence of focus field superposition was observed and published once by Kurt et al.^32^.

The feasibility of Doppler-MPS when three-dimensional space is sampled by the FFP was shown via simulation. The splitting of the higher harmonics was visualized by showing the frequency component resulting from the mixing factors $m_\text{x}$ = 5, $m_\text{y}$ = 0 and $m_\text{z}$ = 0 (Fig. 5). To avoid misinterpretation of split peaks that are caused by the tracer distribution, averaging over many harmonics is recommended for velocity quantification. Besides sampling the volume with a 3D-Lissajous trajectory, the volume could possibly be scanned line by line or radial, which would not necessarily lead to frequency mixing.

In addition, it was shown that the frequency shift of the signal induced into the Rx-channel for a specific particle velocity $v_{\mathrm{s} \, \mathrm{x}}$ determined by 3D-Lissajous sampling is decreased compared to a 1D excitation scheme. A possible explanation for this is presented in supplementary Fig. S1a and b. The simulation shows that the frequency shift decreases with increasing distance $y_\text{sample}$ between the parallel 1D FFP and sample trajectory. On a 3D-Lissajous trajectory, the FFP always passes the particle sample in a certain distance unequal to zero. This explains the decreased frequency shift obtained by a 3D-Lissajous excitation. There is only a very minor impact of the distance $y_\text{sample}$ on the frequency shift in case of 3D-space sampling. This is important for correct velocity quantification without exact knowledge of the particles’ spatial position.

To provide the proof of principle of the presented approach, an experimental setup was developed that mimics linear, uniform motion of SPIONs. The mechanical assembly enabled the oscillation of a SPION sample inside the mf-MPI bore with a nearly uniform motion in the boundaries of the FOV. With the use of the developed assembly, the peak splitting of the higher harmonics due to particle motion could be shown experimentally (Fig. 4b).

Doppler-MPS flow velocity quantification of a SPION sample has been performed successfully. The calibration of the frequency shifts as a function of the particle velocity was performed in simulation. Therefore, the same parameters as used in the experiments have been set in the simulations (drive field waveform, frequency and amplitude, gradient strength, particle properties). The flow velocity quantifications showed a good match between the simulated frequency shifts and the frequency shifts determined from the measurements, since a good mean accuracy was achieved (5 kHz: 0.76 ± 0.31 $\%$, 2 kHz: 1.86 ± 1.24 $\%$). Furthermore, the linear regressions of the quantified velocities showed only very small deviations from slope one and y-intercept zero, which would be a perfect match (5 kHz: $v = 0.987 v_\text{cal} + 0.007$, 2 kHz: $v = 1.008 v_\text{cal} - 0.019$). The correlation coefficients $r^2$ are almost one for both excitation frequencies (5 kHz: $r^2 = 0.9999$, 2 kHz: $r^2 = 0.9992$), which means there is an almost perfect linear correlation. Flow velocity quantifications were presented for sample velocities between 0.28 m/s and 2.07 m/s. It has to be noted that the upper limit is only set by the stability of the mechanical assembly. The lowest measurable velocity was limited by the friction in the mechanical assembly, meaning the DC motor only started to rotate for input voltages equal to or greater than 0.96 V. Thus, velocities lower than 0.28 m/s could not be set.

The measurements of the phantom comprising three SPION samples show that the presented approach is able to quantify flow velocities of particle distributions other than delta samples or boluses. Again, a good mean accuracy (1.80 ± 0.59 $\%$) and a linear regression ($v = 0.987 v_\text{cal} - 0.008$) with only small deviation to slope one and y-intercept zero was achieved (Fig. 7, bottom). Furthermore, the correlation coefficient $r^2$ is almost one (0.9991). However, it can be observed that the standard deviations of the separate velocity quantifications are slightly increased, in particular for higher sample velocities. A reason might be the decreasing SNR of higher harmonics for particle distributions that are more evenly spread across the FOV. The more evenly the particle distribution is spread over the FOV, the stronger the drop of higher harmonics in frequency space will be until only the fundamental harmonic remains in case of a steady state flow, i.e. a homogeneous particle distribution along the direction of flow. Finally, the frequency shift cannot be detected any longer. It has to be noted that image domain velocity quantification is not able to quantify steady state flow velocities, too.

Kaul et al. quantified flow velocities up to 0.21 m/s by bolus tracking in a vessel phantom and further performed in-vivo flow velocity measurements in a mouse model^22^. The linear regression ($v = 1.000 v_\text{cal} - 0.04$) of their estimated flow velocities shows that the method works well in a phantom and in the mentioned flow regime. In-vivo, they measured 48 ± 11 mm/s in the vena cava. A phase contrast MRI measurement revealed a velocity of 40 ± 15 mm/s. Measurements of flow velocities above 0.21 m/s were not shown. For increasing velocities, temporal blurring will become more prominent, which might lead to an inaccuracy in bolus arrival time estimation and hence, a reduced accuracy in velocity quantification. Recently, Franke et al. measured peak flow velocities up to 1.0 m/s exploiting pulsed tracer information^26^ and Vogel et al. showed images of boluses moving with up to 0.4 m/s in their traveling wave MPI scanner^27^.

Flow velocities up to 2.07 m/s were quantified in this work, which has not been be provided by MPI bolus tracking yet. This enables velocity quantifications in the range of physiological flows, as they exceed 0.5 m/s at peak in murine and even 1 m/s in human ascending aorta^33–36^ and carotid arteries^8,37^, which play a crucial role in the formation of stroke. With respect to in-vivo measurements in mice, the lower velocity limit has to be slightly reduced to quantify flow velocities in the vena cava (<0.05 m/s in average)^22,23^. As already stated, the lowest measured velocity in this work (0.28 m/s) was limited by the experimental setup. An advanced setup would enable the quantification of even lower velocities, until the lower detectable velocity limit will be due to spectral resolution, since peak splitting must be visible.

Besides increasing the spectral resolution or using even higher harmonics (with sufficiently high SNR), to further decrease the lower detectable velocity limit, one could process the raw data, such that only the peak shift in one direction shows up in the frequency spectrum. This could be achieved e.g. by DC filtering, followed by cutting the upper or lower half of the raw voltage data. As a result, the constraint by peak splitting is not valid anymore, and one could only observe the shifted peak.

It has to be noted that the aforementioned bolus tracking techniques^22–27^ use 3D FFP trajectories for image acquisitions, whereas 1D excitation is applied in the presented experiments. Since the repetition time of a 1D excitation scheme is considerably lower, according to Eq. (1), bolus tracking with 1D excitation would also be able to quantify considerably higher velocities. Additionally, for 1D excitation, Eq. (1) is a limitation for Doppler-MPS too, since at least two FFP oscillations are needed to obtain a periodic signal. However, in case of multi-dimensional drive field, a periodic signal is already received during one repetition. Thus, the frequency shift including the velocity information is already encoded in the signal of one repetition, whereas the detectable velocity range of bolus tracking is still restricted by the repetition time. According to Eq. (1) and the parameters that were set in the simulation experiment shown in Fig. 5, the maximum velocity that can be detected with bolus tracking would be $v^\text{max}$ = 0.53 m/s, whereas supplementary Fig. S1c shows that Doppler-MPS is able to quantify higher velocities even when 3D-space is scanned (shown for up to 2.6 m/s). The upper limit of detectable velocity could be increased by performing image reconstruction on subsets of a full trajectory, however, other issues as decreased image quality would probably come along with this. In case of Doppler-MPS, the upper detectable velocity limit is reached when the sample is too fast to generate sufficient SNR. For very high velocities, the shifted peaks of specific frequency components will start to overlap, thus, the detection of the frequency shift will become difficult.

Concerning the medical application of the presented technique, the spatial encoding of the velocity needs to be discussed. As the principle of signal generation in Doppler-MPS is equal to conventional magnetic particle imaging or bolus tracking, the spatial information is encoded in the raw data. Thus, regular image reconstruction can be performed to generate images and search for the moving bolus or particle distribution. At a certain bolus speed, temporal blurring will probably be an issue, however, likewise bolus tracking will suffer from that. When the particle distribution cannot be imaged properly any more, co-registration and image fusion with other morphological imaging modalities as CT or MRI might help to define a volume of interest (VOI) for the velocity quantification with Doppler-MPS. The applied FFP trajectory can then be adapted in size and aligned to the defined VOI by use of focus fields and adaptation of the drive fields and/or the selection field. Thus, the quantified velocity can be constrained to the defined VOI.

Another aspect that might be clinically relevant is the detection of different velocities within the scanned FOV. An exemplary simulation experiment was performed to show the ability of Doppler-MPS to quantify various velocities inside a multi-dimensional FOV by scanning the space line by line. The simulated signals in time and frequency space as well as the quantified velocities by each line scan (1D FFP oscillation) are shown in the supplementary Fig. S3. The direction of 1D FFP oscillation is aligned along the direction of flow, otherwise the single line scans will deliver the respective velocity components of the tracer distribution across the FFP trajectory, which potentially will not be separable in frequency space. There is a slight deviation between the quantified velocity and the velocity of the sample at $y_{\mathrm{FFP}}$ (max. 5.8 $\%$) that stems from the fact that the signal of the other samples is detected as well and leads to a superposition of the signal peaks. Finally, four different velocities ($\lbrace$0.5, 1, 1.5, 2$\rbrace$ m/s) are quantified in a two-dimensional FOV (11.2 $\times$ 6.0 mm$^2$) by an exemplary simulation experiment.

## Conclusion

A novel approach to quantify flow velocities based on the principle of MPI has been presented. The method exploits the velocity-induced frequency shift of the emitted tracer signal. The concept of Doppler-MPS was described and the frequency shift as a function of the tracer velocity was derived for one-dimensional drive field. The theoretically described effects were successfully validated by simulations and measurements of a moving SPION sample, thus, a proof of principle was provided. Measurements of a phantom comprising three SPION samples showed the ability to quantify the velocity of particle distributions other than simple boluses. The feasibility of Doppler-MPS when 3D-space is scanned was shown via simulation. The range of measurable velocities achieved with the presented approach enables in-vivo velocity quantification of blood flow in human and murine ascending aorta as well as in human carotid artery and exceeds the maximum velocity that has been quantified by existing MPI-based velocity reconstruction techniques yet.

Since the principle of Doppler-MPS is compatible with conventional MPI, the method enables the combination of velocimetry and imaging in a single device. Quantifying flow velocities of extended range with high accuracy together with being sensitive, fast, depth- and angle-independent and without ionizing radiation underlines the potential of the presented approach for future clinical applications.

## Methods

### Derivation of frequency shift

Magnetic particle imaging measures the magnetization of the SPIONs ${{\varvec{M}}}(t)$ as a response to the external magnetic field ${{\varvec{H}}}({{\varvec{r}}},t)$, which varies in time *t* and space ${{\varvec{r}}}$. In case of 1D encoding in *x*-direction, the external field is a superposition of the dynamic drive field $H_\text{D}(t)$ and the static selection field $H_\text{S}(x)$. The external magnetic field can be written as

10 $$\begin{aligned} H(x,t) = H_{\mathrm{s}}(x) + H_\text{D}(t) . \end{aligned}$$

At position $x = 0$, the selection field $H_{\mathrm{s}} (x) = Gx$ vanishes, where *G* is the gradient strength. The drive field can be written as $H_\text{D}(t) = G \, x_{\mathrm{FFP}}(t)$, with the trajectory of the field free point $x_{\mathrm{FFP}}(t)$. Then, the external field can be expressed as

11 $$\begin{aligned} H(t) = H_\text{D}(t) = G \, x_{\mathrm{FFP}}(t). \end{aligned}$$

Considering a delta distribution of SPIONs in the laboratory system that performs uniform linear motion in *x*-direction with the velocity $v_{\mathrm{s}}$, the FFP trajectory $x_{\mathrm{FFP}}^{\prime }(t)$ in the reference frame of the SPIONs is modulated (c.f. Eq. (2)). The modulation of the FFP trajectory in the SPIONs’ reference system $x_{\mathrm{FFP}}^{\prime }(t)$ caused by the particles’ motion in the laboratory system is illustrated in Fig. 2.

At first, the special case of ideal particles is observed. Here, the term *ideal* refers to a particles’ magnetization behavior according to the sign function (c.f. Eq. (7)):

12 $$\begin{aligned} M(H) \propto \mathrm{sgn}(H) \end{aligned}$$

In the reference frame of the particles, at position $x^{\prime } = 0$, which is the position of the particles in the laboratory system, the external magnetic field is $H^{\prime }(t) = G \, x_{\mathrm{FFP}}^{\prime }(t)$. Thus, the particles’ magnetization can be expressed as

13 $$\begin{aligned} M(t) \propto \mathrm{sgn}\left( G \, x_{\mathrm{FFP}}^{\prime }(t)\right) = \mathrm{sgn}\left( G \left( x_{\mathrm{FFP}}(t) + v_{\mathrm{s}} t \right) \right) . \end{aligned}$$

In MPI, the SPIONs’ magnetization is measured by receive coils, in which voltage is induced due to magnetization change. Thus, the measured particle signal *S*(*t*) is proportional to the time derivative of the magnetization. Consequently, the signal can be written as follows:

14 $$\begin{aligned} S(t) \propto \frac{\partial }{\partial t} M(t) = 2G \, \frac{\partial }{\partial t} x_{\mathrm{FFP}}^{\prime }(t) \, \delta (H^{\prime }(t)), \end{aligned}$$

where $\delta (H)$ is the Dirac delta function. As a consequence, the signal is only unequal to zero, when $H^{\prime }(t) = 0$. Hence, the delta peaks in time space appear at zero crossings of the modulated FFP trajectory, which is illustrated in Fig. 3a,b, where ideal particles are observed.

The distance between the signal peaks in time space can be interpreted as the period time $T_{\mathrm{s}}$ of the signal’s fundamental harmonic. Thus, the frequency of the signal’s fundamental harmonic can be calculated by $\omega _{\mathrm{s}} = 2\pi / T_{\mathrm{s}}$ with $\omega = 2 \pi f$.

#### Ideal particles, triangular excitation

For the purpose of calculating the roots of the modulated FFP trajectory $x_{\mathrm{FFP}}^{\prime }(t)$, the case of triangular excitation

15 $$\begin{aligned} x_{\mathrm{FFP}}(t) = x_{\mathrm{FFP}}^\text{max} \, \mathrm{tri} (\omega _\text{D} t) \end{aligned}$$

is considered first, where $x_{\mathrm{FFP}}^\text{max}$ is the amplitude of the field free point trajectory and $\omega _\text{D}$ is the drive field frequency. At $x_{\mathrm{FFP}}^{\prime }=0$, one can write

16 $$\begin{aligned} x_{\mathrm{FFP}}^{\prime }(t) = \pm 2 \, x_{\mathrm{FFP}}^\text{max} \, \frac{\omega _\text{D}}{\pi } \, \left( t - \frac{ 2 \pi }{\omega _\text{D}} \, n \right) + v_{\mathrm{s}} t , \end{aligned}$$

with $n \in \mathbb {N}$. Solving Eq. (16) for *t* leads to the points in time of zero crossings:

17 $$\begin{aligned} t = 4 \, x_{\mathrm{FFP}}^\text{max} \, n \, \left( 2 x_{\mathrm{FFP}}^\text{max} \frac{\omega _\text{D}}{\pi } \pm v_{\mathrm{s}}\right) ^{-1} \end{aligned}$$

Note that one gets two solutions according to increasing, respectively decreasing FFP trajectory. The particle signal’s period time and fundamental frequency finally are

18 $$\begin{aligned} T_{\mathrm{s}} = 4 \, x_{\mathrm{FFP}}^\text{max} \, \left( 2 \, x_{\mathrm{FFP}}^\text{max} \, \frac{\omega _\text{D}}{\pi } \pm v_{\mathrm{s}}\right) ^{-1} \end{aligned}$$

and

19 $$\begin{aligned} \omega _{\mathrm{s}} = \frac{\pi }{2 \, x_{\mathrm{FFP}}^\text{max}} \, \left( 2 \, x_{\mathrm{FFP}}^\text{max} \, \frac{\omega _\text{D}}{\pi } \pm v_{\mathrm{s}}\right) = \omega _\text{D} \pm \frac{\pi }{2 \, x_{\mathrm{FFP}}^\text{max}} \, v_{\mathrm{s}} \, , \end{aligned}$$

where $\Delta \omega = (\pi / 2 \, x_{\mathrm{FFP}}^\text{max}) \, v_{\mathrm{s}}$ is the frequency shift, which is independent of the drive field frequency $\omega _\text{D}$. Since one gets two solutions for the shifted frequency of the signal’s fundamental harmonic $\omega _{\mathrm{s}} = \omega _\text{D} \pm \Delta \omega$, the harmonics in frequency space split up into two peaks.

The FFP trajectory $x_{\mathrm{FFP}}^{\prime }(t)$, the particles’ magnetization *M*(*t*) and the signal *S*(*t*) are depicted in Fig. 3a. The frequency shift $\Delta f = \Delta \omega / 2 \pi$ against the particles’ velocity is shown in Fig. 3d for an exemplary FFP trajectory amplitude $x_{\mathrm{FFP}}^\text{max} = 12$ mm.

#### Ideal particles, sinusoidal excitation

In order to calculate the roots of the modulated FFP trajectory for the case of sinusoidal excitation

20 $$\begin{aligned} x_{\mathrm{FFP}}(t) = x_{\mathrm{FFP}}^\text{max} \, \mathrm{sin} \left( \omega _\text{D} t\right) \end{aligned}$$

the sine function can be approximated by the Taylor series

21 $$\begin{aligned} \sin (x) \approx x - \frac{x^3}{6} + \frac{x^5}{120} - \, \,... \, , \end{aligned}$$

which is a good approximation near the zero crossings, if one expresses the FFP trajectory $x_{\mathrm{FFP}}^{\prime }(t)$ as

22 $$\begin{aligned} x_{\mathrm{FFP}}^{\prime }(t) = \pm \, x_{\mathrm{FFP}}^\text{max} \, \left( \omega _\text{D} \, t_n - \frac{\left( \omega _\text{D} \, t_n\right) ^3}{6} + \frac{\left( \omega _\text{D} \, t_n\right) ^5}{120} - \, ... \right) + v_{\mathrm{s}} t \, , \end{aligned}$$

where $t_{n} = t - \frac{ 2 \pi }{\omega _\text{D}} \, n$. Again, the plus-sign refers to increasing and the minus-sign refers to decreasing FFP trajectory. The roots of Eq. (22) that provide information on when the delta peaks appear in the signal were computed and the shifted frequency was calculated based on the first signal period $T_{\mathrm{s}} = t_1 - t_0$, since there is a slight shift in the period time $T_{\mathrm{s}}$ with increasing *n*. Figure 3d shows the shifted fundamental frequency $\Delta f$ dependent on the particles’ velocity, assuming a sine approximation with the Taylor series up to the fifth order. For the case of a sinusoidal drive field, the FFP trajectory $x_{\mathrm{FFP}}^{\prime }(t)$, the particles’ magnetization *M*(*t*) and the signal *S*(*t*) are depicted in Fig. 3b.

#### Langevin particles, sinusoidal excitation

Assuming a particles’ magnetization behavior according to the Langevin theory, the magnetization *M*(*t*) of a SPION delta distribution can be calculated according to Eq. (4). As already mentioned above, the particle signal is proportional to the time derivative of the magnetization: $S(t) \propto \partial /\partial t \, M(t)$. The signal *S*(*t*) as well as the FFP trajectory $x_{\mathrm{FFP}}^{\prime }(t)$ and the particles’ magnetization *M*(*t*) is shown in Fig. 3c.

To find the periodicity of the signal, one can again interpret the distance between two signal peaks (two local maxima, respectively minima) as period time $T_{\mathrm{s}}$. The time points of the signal maxima and minima were found by calculating the roots of the second derivative of the magnetization $\partial ^2/\partial t^2 M(t)$. The shifted fundamental frequency $f_{\mathrm{s}}$ was calculated according to the first period of the signal against the particles’ velocity $v_{\mathrm{s}}$ by $f_{\mathrm{s}} =1/T_{\mathrm{s}}$.

The fundamental frequency shift $\Delta f$ against the particles’ velocity $v_{\mathrm{s}}$ is shown in Fig. 3d. A magnetic moment of $m_0 = 3.35 \cdot 10^{-18}$ Am$^2$ was assumed, which was estimated from a core diameter of 20 nm and a saturation magnetization of 800 kA/m. The gradient strength *G* was set to 1 T/m and room temperature was assumed.

### Calibration of frequency shift

To quantify the velocities, the knowledge of the frequency shift as a function of the sample velocity is required. This information can be taken from the theoretical model or can be determined by a calibration either in simulation or in an experiment. Here, the calibration was performed in simulation, since the frequency shifts $\Delta f_\text{n}$ could be taken directly from the higher harmonics in the FFT of the particle signal. The shifted harmonics $f_{\mathrm{s} \, \mathrm{n}}$ in the simulated frequency spectra were determined by gaussian fitting of each peak and choosing the maxima as the shifted frequency. The frequency shift of the fundamental frequency $\Delta f$ can then be calculated according to $\Delta f = \Delta f_\text{n} / n$ with $n \in \mathbb {N}$. This can be done for a number of higher harmonics to get an averaged shift of the fundamental frequency. The calibration was performed for sample velocities between 0 m/s an 2.6 m/s with the use of same parameters as set in the experiments (drive field waveform, frequency and amplitude, gradient strength, particle properties). The calibration was saved as coefficients of linear regression of the fundamental harmonic’s frequency shifts as a function of the sample velocity.

### Quantification of sample velocity

For quantification of the sample velocity, the shifted fundamental frequency $f_{\mathrm{s}}$ is determined from a number of higher harmonics of the measured signal according to $\Delta f = \Delta f_\text{n} / n$ by gaussian fitting of the peaks. For the experimental velocity quantification, the harmonics from number 4 to 7 were chosen to determine the shifted fundamental harmonic $f_{\mathrm{s}}$, since the divided peaks could be distinguished well and showed sufficiently high SNR. After averaging the value for the shifted fundamental harmonic, the particle velocity finally can be taken directly from the calibration curve.

## Supplementary Information

Supplementary Information.

## Data Availability

The authors declare that the data supporting the findings of this study are available within the manuscript. The raw data of simulation, calibration and velocity measurements are available from the corresponding author upon reasonable request.

## References

[1] James SL (2018). Global, regional, and national incidence, prevalence, and years lived with disability for 354 diseases and injuries for 195 countries and territories, 1990–2017: A systematic analysis for the Global Burden of Disease Study 2017. Lancet.

[2] van de Hoef TP (2012). Coronary pressure-flow relations as basis for the understanding of coronary physiology. J. Mol. Cell Cardiol..

[3] Memmola C (1994). Coronary flow dynamics and reserve assessed by transesophageal echocardiography in obstructive hypertrophic cardiomyopathy. Am. J. Cardiol..

[4] Schwitter J (2000). Valvular heart disease: Assessment of valve morphology and quantification using MR. Herz.

[5] Atherton JJ, Moore TD, Thomson HL, Frenneaux MP (1998). Restrictive left ventricular filling patterns are predictive of diastolic ventricular interaction in chronic heart failure. J. Am. Coll. Cardiol..

[6] Taylor CA (1999). Predictive medicine: Computational techniques in therapeutic decision- making. Comput. Aided Surg..

[7] Sankaran S (2012). Patient-specific multiscale modeling of blood flow for coronary artery bypass graft surgery. Ann. Biomed. Eng..

[8] Nederkoorn PJ, Van Der Graaf Y, Hunink MG (2003). Duplex ultrasound and magnetic resonance angiography compared with digital subtraction angiography in carotid artery stenosis: A systematic review. Stroke.

[9] Bonnefous O (2012). Quantification of arterial flow using digital subtraction angiography. Med. Phys..

[10] Thompson RB, McVeigh ER (2002). High temporal resolution phase contrast MRI with multiecho acquisitions. Magn. Resonan. Med..

[11] Lotz J, Meier C, Leppert A, Galanski M (2002). Cardiovascular flow measurement with phase-contrast MR imaging: Basic facts and implementation. Radiographics.

[12] Gatehouse PD (2005). Applications of phase-contrast flow and velocity imaging in cardiovascular MRI. Eur. Radiol..

[13] Franklin DL, Schlegel W, Rushmer RF (1961). Blood flow measured by Doppler frequency shift of back-scattered ultrasound. Science.

[14] Gleich B, Weizenecker J (2005). Tomographic Imaging using the Nonlinear Response of Magnetic Particles. Nature.

[15] Weizenecker J, Gleich B, Rahmer J, Dahnke H, Borgert J (2009). Three-dimensional real-time in vivo magnetic particle imaging. Phys. Med. Biol..

[16] Knopp T, Gdaniec N, Möddel M (2017). Magnetic particle imaging: From proof of principle to preclinical applications. Phys. Med. Biol..

[17] Vogel P (2019). Micro-traveling wave magnetic particle imaging—sub-millimeter resolution with optimized tracer LS-008. IEEE Trans. Magn..

[18] Tay ZW (2019). Pulsed excitation in magnetic particle imaging. IEEE Trans. Med. Imag..

[19] Graeser M (2017). Towards picogram detection of superparamagnetic iron-oxide particles using a gradiometric receive coil. Sci. Rep..

[20] Zheng B (2015). Magnetic particle imaging tracks the long-term fate of in vivo neural cell implants with high image contrast. Sci. Rep..

[21] Wang P (2018). Magnetic particle imaging of islet transplantation in the liver and under the kidney capsule in mouse models. Quant. Imaging Med. Surg..

[22] Kaul MG (2018). Magnetic particle imaging for in vivo blood flow velocity measurements in mice. Phys. Med. Biol..

[23] Jung CMPI (2016). Biomedical applications in molecular. Struct. Funct. Imaging.

[24] Sedlacik J (2016). Magnetic particle imaging for high temporal resolution assessment of aneurysm hemodynamics. PLoS ONE.

[25] Siepmann, R. *et al.* MPI velocity mapping in a coronary vessel phantom. In *International Workshop of Magnetic Particle Imaging* (2019).

[26] Franke J (2020). Hybrid MPI-MRI system for dual-modal in situ cardiovascular assessments of real-time 3D blood flow quantification—a pre-clinical in vivo feasibility investigation. IEEE Trans. Med. Imaging.

[27] Vogel P (2020). Superspeed bolus visualization for vascular magnetic particle imaging. IEEE Trans. Med. Imaging.

[28] Doppler C (1842). Über das farbige Licht der Doppelsterne und einiger anderer Gestirne des Himmels. Abh. Königl. Böhm. Ges. Wiss..

[29] Bedanta S, Kleemann W (2009). Supermagnetism. J. Phys. D Appl. Phys..

[30] Pantke D, Holle N, Mogarkar A, Straub M, Schulz V (2019). Multifrequency magnetic particle imaging enabled by a combined passive and active drive field feed-through compensation approach. Med. Phys..

[31] Lawaczeck R (1997). Magnetic iron oxide particles coated with carboxydextran for parenteral administration and liver contrasting: Pre-clinical profile of SH U555A. Acta Radiol..

[32] Kurt S, Abdulla V, Saritas EU (2020). Multi-dimensional harmonic dispersion x-space MPI. Int. J. Magn. Particle Imaging.

[33] Angelsen BA, Brubakk AO (1976). Transcutaneous measurement of blood flow velocity in the human aorta. Cardiovasc. Res..

[34] Gardin JM, Burn CS, Childs WJ, Henry WL (1984). Evaluation of blood flow velocity in the ascending aorta and main pulmonary artery of normal subjects by Doppler echocardiography. Am. Heart J..

[35] Gisvold SE, Brubakk AO (1982). Measurement of instantaneous blood-flow velocity in the human aorta using pulsed Doppler ultrasound. Cardiovasc. Res..

[36] Hartley CJ, Michael LH, Entman ML (1995). Noninvasive measurement of ascending aortic blood velocity in mice. Am. J. Physiol. Heart Circ. Physiol..

[37] Pomella N (2017). Common carotid artery diameter, blood flow velocity and wave intensity responses at rest and during exercise in young healthy humans: A reproducibility study. Ultrasound Med. Biol..

