# Supplementary material for: Flow velocity quantification by exploiting the principles of the Doppler effect and magnetic particle imaging
Source: Sci Rep. 2021 Feb 25;11:4529. doi: 10.1038/s41598-021-83821-w (PMC7907137; doi:10.1038/s41598-021-83821-w)
Supplement: Supplementary file 1 — Supplementary Information. [file 41598_2021_83821_MOESM1_ESM.pdf]

Supplementary material

Flow velocity quantification by exploiting the principles of the Doppler effect and magnetic particle imaging

Dennis Pantke[1,*], Florian Mueller[1], Sebastian Reinartz[3], Fabian Kiessling[2,4], and Volkmar Schulz[1,4,5,*]

[1]Department of Physics of Molecular Imaging, Institute for Experimental Molecular Imaging, RWTH Aachen University, Aachen, Germany
[2]Institute for Experimental Molecular Imaging, Medical Faculty, RWTH Aachen University, Aachen, Germany
[3]Department of Diagnostic and Interventional Radiology, Uniklinik RWTH Aachen, Aachen, Germany
[4]Fraunhofer Institute for Digital Medicine MEVIS, Bremen, Germany
[5]III. Physikalisches Institut B, RWTH Aachen University, Aachen,Germany
[*]{dennis.pantke,volkmar.schulz}@pmi.rwth-aachen.de

ABSTRACT

Changes in blood flow velocity play a crucial role during pathogenesis and progression of cardiovascular diseases. Imaging techniques capable of assessing flow velocities are clinically applied but are often not accurate, quantitative, and reliable enough to assess fine changes indicating the early onset of diseases and their conversion into a symptomatic stage. Magnetic particle imaging (MPI) promises to overcome these limitations. Existing MPI-based techniques perform velocity estimation on the reconstructed images, which restricts the measurable velocity range. Therefore, we developed a novel velocity quantification method by adapting the Doppler principle to MPI. Our method exploits the velocity-dependent frequency shift caused by a tracer motion-induced modulation of the emitted signal. The fundamental theory of our method is deduced and validated by simulations and measurements of moving phantoms. Overall, our method enables robust velocity quantification within milliseconds, with high accuracy, no radiation risk, no depth-dependency, and extended range compared to existing MPI-based velocity quantification techniques, highlighting the potential of our method as future medical application.

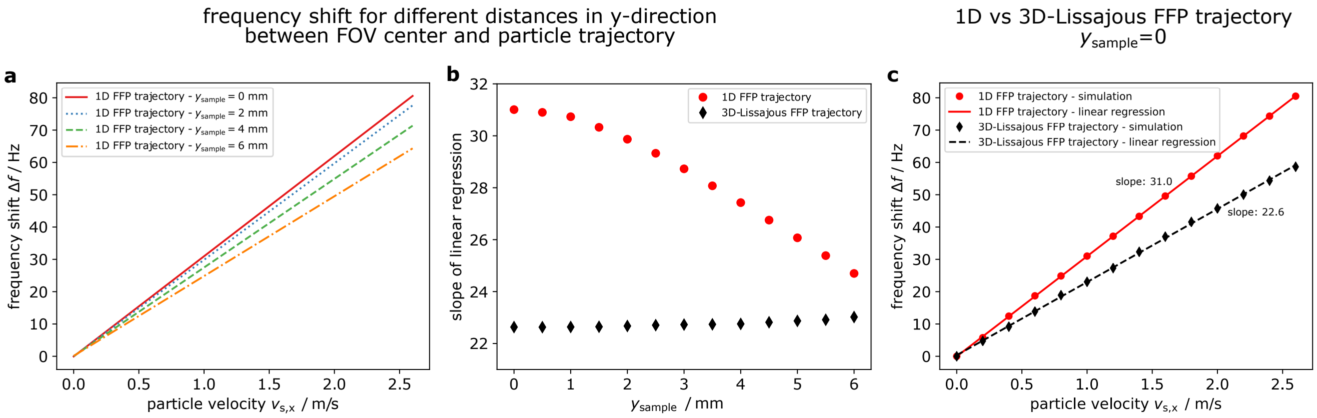

Figure S1. Simulated frequency shifts for different distances between FOV center and sample trajectory and simulated frequency shift for 1D and 3D-Lissajous FFP trajectory: a) Frequency shift against particle velocity for different distances y_{sample} between parallel trajectories of particle sample and FFP. b) The slope of linear regressions of the frequency shift for different distances y_{sample} for 1D and 3D-Lissajous trajectory, where $y_{\text{sample}} = 0$ is the FOV center. c) Simulated frequency shift for 1D vs 3D-Lissajous FFP trajectory at $y_{\text{sample}} = 0$. The drive field frequencies that generate the Lissajous trajectory are $f_x = 24.5098$ kHz, $f_z = 26.04167$ kHz and $f_z = 25.2525$ kHz, whereas the drive field frequency for the 1D FFP trajectory simulation is f_x. All simulations were performed with the following set of parameters: $H_{\text{D x,y,z}} = 14\ \text{mT}/\mu_0$, $G_x = 2.5$ T/m, $G_{y,z} = \frac{1}{2}G_x$, $m_0 = 3.35 \cdot 10^{-18}\ \text{Am}^2$. The frequency shift resulting from the 3D-Lissajous trajectory is lower than obtained by 1D simulation. The frequency shift was determined by analyzing the frequency components resulting from the mixing factors $m_x = \{4, 5, 6\}$, $m_y = 0$, $m_z = 0$ and averaging the respective frequency shift.

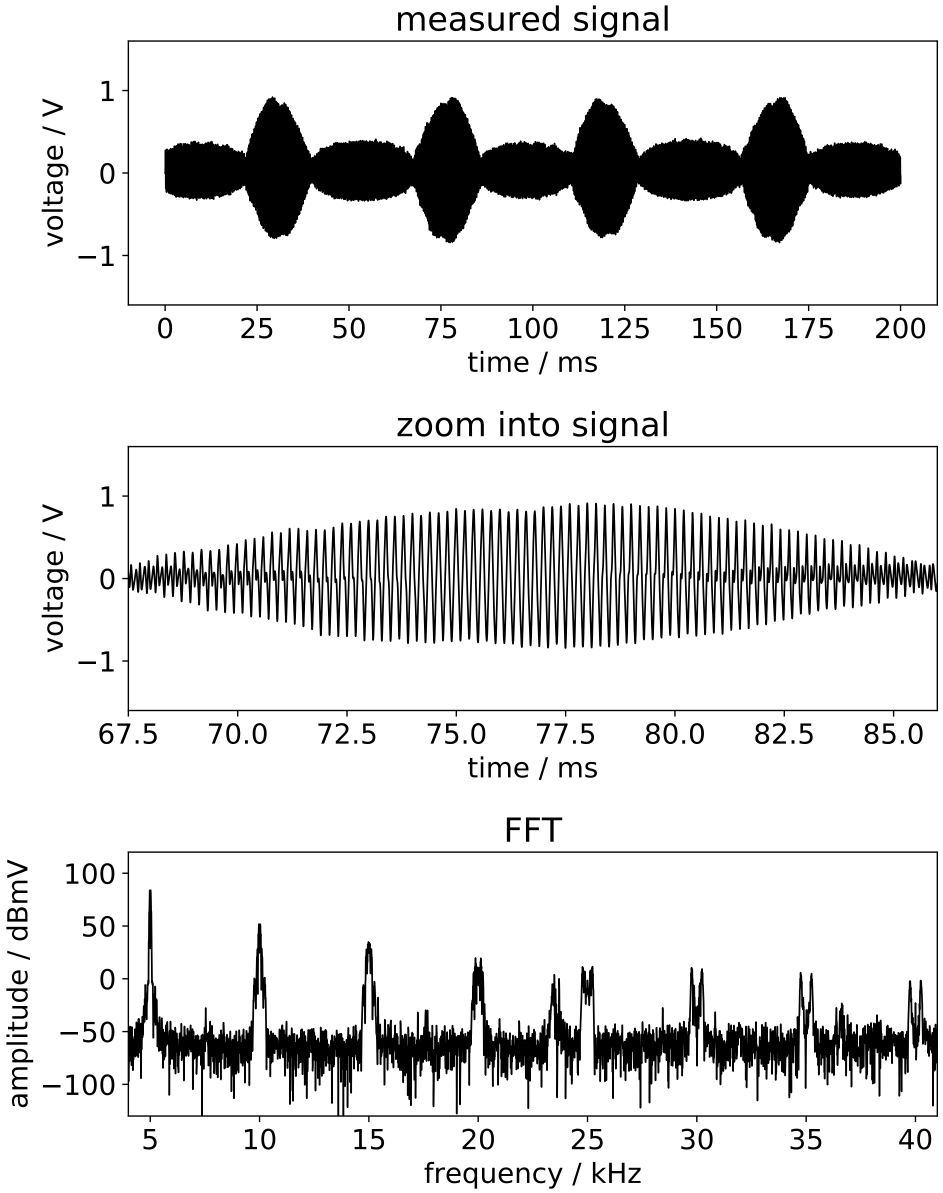

Figure S2. Measured signal of 3-sample phantom in time and frequency domain: Measured signal of the oscillating 3-sample phantom filled with 60 μl Perimag each, in time (top, center) and frequency space (bottom). The signal is shown exemplarily for an excitation frequency of 5 kHz and a quantified sample velocity of 2.03 m/s. In time domain, four field of view (FOV) crossings are shown (top) and a zoom into one FOV crossing is provided (middle). To obtain the signal in frequency space, the Fourier transform of the signal caused by one FOV crossing is performed. The splitting of the higher harmonics is visible in the frequency spectrum, particularly at high harmonic numbers.

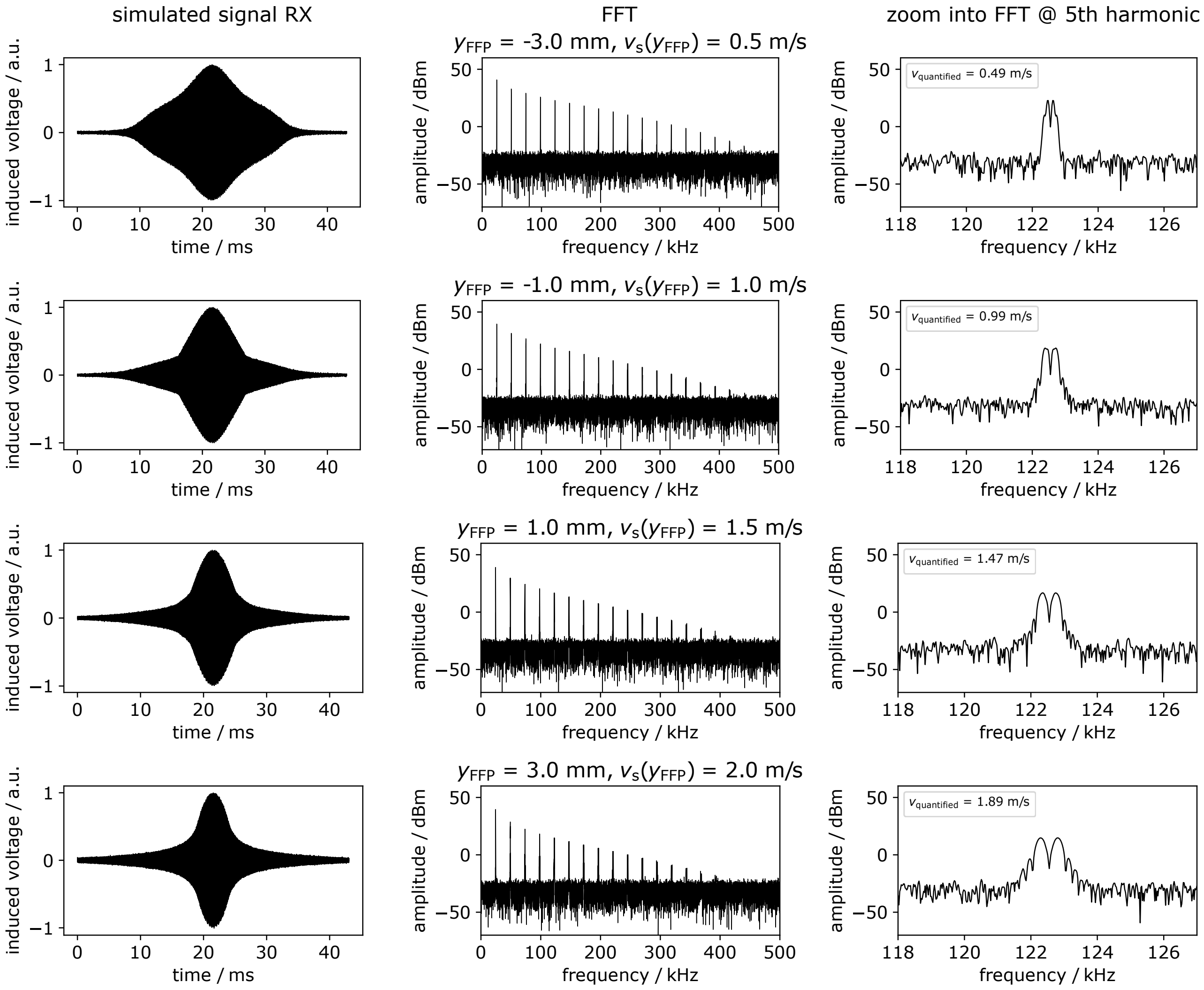

Figure S3. Line-by-line scan of four moving samples inside the FOV with varying velocities. Rx-channel signal of four simultaneously moving samples with varying velocities in x-direction in time (left) and frequency domain (center, right). The velocity distribution is $\{0.5, 1.0, 1.5, 2.0\}$ m/s at y_{sample} = {-3, -1, 1, 3} mm. Four line scans are shown with $y_{\text{FFP}} = y_{\text{sample}}$, such that the FFP trajectory and the respective sample motion line are in parallel. The drive field frequency is f_x = 24.5098 kHz. Other relevant simulation parameters are $H_{\text{Dx,y,z}} = 14$ mT$/\mu_0$, G_x = 2.5 T/m, $G_y = \frac{1}{2}G_x$, $x_{\text{FFP}}^{\text{max}}$ = 5.6 mm, $m_0 = 3.35 \cdot 10^{-18}$ Am^2, T = 293 K. Different velocities inside the FOV can be detected. The quantified velocities are shown in the right images. The deviation between the quantified velocity and the velocity of the sample at y_{FFP} stems from the fact that the signal of the other samples is detected as well and leads to a superposition of the signal peaks. This finally leads to a small shift of the resulting peak.
